# *Manzuaat wa Musharadat*, Uprooted and Scattered: Refugee Women Escape Journey and the Longing to Return to Syria

**DOI:** 10.3389/fpsyg.2021.537131

**Published:** 2021-02-02

**Authors:** Niveen Rizkalla, Suher Adi, Nour Khaddaj Mallat, Laila Soudi, Rahma Arafa, Steven P. Segal

**Affiliations:** ^1^School of Social Welfare, University of California, Berkeley, Berkeley, CA, United States; ^2^Political Science & Middle Eastern Studies, University of California, Berkeley, Berkeley, CA, United States; ^3^School of Public Health, University of California, Berkeley, Berkeley, CA, United States; ^4^School of Medicine, Stanford University, Stanford, CA, United States

**Keywords:** refugees, women, escape journey, Syrian crisis, longing to return, narratives

## Abstract

**Objective:**

Violent conflict forced millions of Syrians to flee their homes to host countries. This study examines Syrian refugee women’s experiences from the war’s outset through their journey to Jordan. It addresses the toll this journey had on their lives.

**Methods:**

Twenty-four in-depth interviews were completed with Syrian refugee women who currently reside in urban areas of Jordan. Researchers translated, transcribed, and analyzed the interviews using group narrative methodology.

**Results:**

The Syrian women had unique nostalgic memories of times before the war. They experienced atrocities during the war that forced their decision to escape Syria. Their journey narratives testify of internal displacement, personal and collective traumatic journeys via legal and illegal routes. Almost all the women were placed in refugee camps during their transitions to host country residency. In Jordan, they faced diverse hurdles of displacement and extremely different realities compared to the ones they had in Syria. Despite how very different but difficult each of their journeys were, every single woman longed to return home to Syria.

**Conclusions:**

This study presents a new understanding of the role and process of the journeys undertaken and highlights the concept of “return” as the defining element for Syrian refugee women. Regardless of the hardships women endured to escape their homeland to find safety, “return” marks an ending to their horror journey and the beginning of a new journey of hope for a better future.

## Introduction

The Syrian war is one of the largest humanitarian tragedies of the 21st century. The vast majority of Syrians were displaced by armed insinuates violence in their country (UNHCR, 2019). Civilian areas were aggressively attacked with missiles and bombs resulting in total destruction. Millions were forcibly displaced, lost their properties and social support systems (Jabbar and Zaza, 2014; Balcilar and Nugent, 2019). They witnessed and experienced traumatic events including combat situations, killings, hunger, physical and sexual assault, as well as torture, and forced separation from family, friends, and communities (Panter-Brick et al., 2018; Rizkalla and Segal, 2018, 2019a). In search of safety, they fled to the neighboring countries and beyond.

Refugees’ escape has been described as a journey of horror. They experienced heavy shelling, freezing weather, hardships in attempting to avoid capture by the Syrian regime, and witnessed the death of their own children or relatives (Sayed Issa, 2017). Some were forced to escape through the mountains (Thorleifsson, 2016), while others undertook diverse routes through the desert (Gillespie et al., 2018), or the Mediterranean Sea (Farhat et al., 2018; Mangrio et al., 2018). The majority experienced internal displacement before reaching the borders (Doocy et al., 2015). During this escape journey, Syrian women and girls were trafficked, sexually abused, raped, or traded for sex slavery (Walton, 2018).

Before reaching the urban areas of Jordan, many Syrians were first hosted in refugee camps in Northern Jordan, mainly in Za’atari camp, Mrajeeb Al Fhood, Cyber City, and Al-Azraq camps (Al-Qdah and Lacroix, 2017). The refugee camps’ poor living conditions and lack of access to healthcare gave rise to preventable infectious diseases (Abbott et al., 2017). Jordanian healthcare officials indicated that serious and highly contagious diseases such as tuberculosis, measles, and cutaneous leishmaniasis were widespread (Murshidi et al., 2013). In the refugee camps, Syrians were unable to afford the costs of continuous medical treatment, often only seeking free treatment for acute illnesses delivered by humanitarian organizations (Abbott et al., 2017). Syrian refugees residing in the camps of Jordan and Turkey reported that the living conditions were unbearable since camps were fenced like prisons, had limited resources, and lacked work opportunities (Gerard and Pickering, 2014; Thorleifsson, 2016).

Newly displaced refugees encounter multiple challenges in the host countries, some of which include housing difficulties, high cost of living, illegality of work, scarce economic resources, poverty, hostility of locals (Achilli, 2015; Carrion, 2015; Verme et al., 2015; Rizkalla et al., 2020a), as well as hyper-attention to the events in Syria and the status of the family members they left behind (Verme et al., 2015; Thorleifsson, 2016). In Jordan, the majority of Syrian refugees live below the poverty line and do not receive basic necessities. In addition to refugees’ displacement challenges, the horrifying war experiences have also triggered serious mental health issues including severe depression, anxiety, and posttraumatic stress disorder (PTSD), as well as physical illnesses and somatic symptomology (Al-Shagran et al., 2015; Abbott et al., 2017; Acarturk et al., 2018; Al Rousan et al., 2018; Rizkalla and Segal, 2018, 2019b; Sagaltici et al., 2019; Rizkalla et al., 2020a,b). Displaced Syrians, especially women, are also subjected to mistreatment and human trafficking by host communities (Canefe, 2018).

The United Nations High Commissioner for Refugees reported Syrian refugees return from Jordan to Syria due to survival difficulties and shortage of food assistance. Eighty-six percent of Syrian refugees who reside in urban areas of Jordan are living in poverty and are forced to beg, drop out of schools and reduce their food consumption for survival (UNHCR, 2015). Some refugees have no choice but to return to Syria seeking medical treatment, despite the ongoing conflict. These refugees are unable to afford the healthcare expenses in Jordan, and therefore travel periodically between Jordan and Syria to seek more affordable treatment in Syria (Gerard and Pickering, 2014; Akik et al., 2019; Balinska et al., 2019).

The complexity of accessing Middle Eastern refugees who are continuously relocating to different geographical destinations makes this research on refugee women’s journeys extremely challenging. Though extensive studies related to the impacts of war and displacement on Syrians exist, first hand women’s journey ordeals are still understudied. This research explores the journey narratives of refugee women from their initial escape from Syria until they reached the host country of Jordan, where they currently reside. Additionally, the study explores a common theme experienced in the larger body of literature on displacement, which is the longing to return.

## Materials and Methods

This study is part of a larger research project on the physical and mental health of Syrian refugees who live in the urban areas of Jordan. The study initially explored a variety of themes disclosed by Syrian refugee women during the war, their subsequent escape journeys, the transition to refugee camps, in addition to displacement challenges. The study focused on understanding the subjective human impacts and realities of Syrian refugee women by providing them with a platform to share their individual and collective experiences utilizing the narrative paradigm in the qualitative research approach (Josselson, 2004; Chase, 2005; Creswell and Poth, 2018). The study focused on psychological narrative inquiry, which looked into emotions, coping mechanisms, and cognitions, with the goal of suggesting a new perspective on displaced women’s journeys.

### Recruitment

The first author created a collaborative relationship with various humanitarian organizations in Jordan. Staff members recruited Syrian women who sought services from organizations to participate in the study and offered them a safe space to share their stories. The participating organizations were Waqea, Jordanian Women’s Union, Bader Center, Naher El-Rahme NGO for Social Development, the Green Crescent Society, Dar El-Karame, and the Charity Organization of the Islamic Center. Gaging refusal to participate in the study was unattainable to the researchers due to the fact that organization staff members were the initial contacts with the refugees in coordinating interviews.

The vast majority of interviews took place during organizations’ working hours; however, some interviews were conducted according to women’s preferences of timing and location, which resulted in interviews conducted at public areas like coffee-shops or at their homes. This approach was utilized to accommodate women who had work commitments, time restraints, or childcare needs. The recruitment process ended following data saturation (Saunders et al., 2018), i.e., at a point in which new interviews were unlikely to add new information.

### Data Collection

Data collection began in March and ended in June 2014. In-depth interviews were conducted in person with the first author in Arabic, and were audio recorded after gaining permission from women prior to their participation. Only one woman feared and refused to be recorded due to the extreme trauma of rape she endured in Syria. The researcher did not record this interview and only took notes in the process. The length of interviews ranged from 40 min to two and a half hours due to the nature of narration. The length of three interviews were in the range of 40 min, another three interviews were in the range from two to two and a half hours, whereas the rest of the interviews were in the range of one hour to one and a half hours. Though political points of view did not dominate the story telling, the national crisis framed all participants’ narratives as a motive for speaking. Criteria for participation were as follows: Syrian refugee woman, at least 19 years old, and currently residing in an urban area in Jordan. Participation did not include incentives, however, all participants were provided with food baskets and blankets by the hosting organizations after the interviews were completed.

The researcher took a semi-structured interview approach in order to explore different aspects of women’s stories and never asked about their political affiliations. Interviews began with obtaining general demographic information. Additional information was shared during the interviews after gaining trust and developing a close relationship with the researcher (Josselson, 2004). Though the researcher began by asking open-ended questions, she allowed a flow in responses (De Fina, 2003), which shifted the interview process from semi-structured to an in-depth ethnographic interview, with a narrative paradigm (Creswell and Poth, 2018). Eventually, all women wanted to share their stories of suffering in the hope that the world would hear and see them; they also acknowledged feeling relief at the end of the process.

Questioning was gradual, beginning with asking about their lives before, during and after the war in Syria, concluding with their conditions in Jordan and their hopes and aspirations for the future. Questions included “How was your life in Syria before the war?” “How did you decide on leaving Syria?” Because women used the terms “escape, flee, and run away,” the following question was asked: “How did you escape and with whom?” Questions later shifted to “How was your experience in the refugee camps or on other routes until you reached the urban areas in Jordan?” Throughout the interviews, the researcher validated her interpretation of women’s narration (Lucius-Hoene and Deppermann, 2000; De Fina, 2003), and took the liberty to ask clarifying questions whenever unfamiliar topics, terms, dialects, or phrases were shared (Clandinin and Connelly, 2000). All interviews were conducted in coordination with cultural practices and provided a familiar welcoming environment. In addition, the researcher was cautious about participants’ wellbeing, and tried not to exacerbate their emotional capacity when reliving traumatic stories. Due to the highly sensitive and emotional nature of the narratives, all interviews concluded with a positive question to help women feel understood and empowered: “What are you hoping for?” and “What aspirations and dreams do you have for the future?”

### Ethics

This research was approved by the Committee for the Protection of Human Subjects, (University of California Berkeley) (CPHS, February 2014). To create a safe environment and ensure truthful responses, only oral consent was required from participants. Prior to participation, each Syrian woman was provided with information, e.g., study’s purposes, procedures, and a consent form. Part of the information that the researcher disclosed was her obligation to report cases of self-harm or risks of self or others’ harm to authorized organizations. Audio recording proceeded after gaining permission. In addition to anonymously, participants were asked to provide pseudonyms of their choice in order to maintain confidentiality for future disseminations when using women’s quotes. Before transcribing and translating the interviews, the audio recordings were labeled by numbers and password-protected.

### Data Analysis

All interviews were transcribed and translated verbatim by a team of four bilingual Arabic and English researchers. Their backgrounds were of Palestinian and Syrian with degrees in mental and public health and social sciences. Translation and transcription of the interviews were divided among the team members equally and were edited by the first author. If a word or phrase was disputed, then the team collectively came to a conclusion on which translation best captured the original meaning. In the final versions of the transcripts, non-verbal cues (e.g., laughter, crying) and setting related-information (e.g., children/staff interruptions, breastfeeding) were added to better convey the atmosphere during the interviews (Clandinin and Connelly, 2000).

The study utilized ethnographic methodology with a narrative paradigm (Creswell and Poth, 2018). The analysis included the content and context of the narratives (De Fina, 2003). In the initial phase, each team member independently detected major repetitive themes. In group meetings, discussions took place to finalize the themes that captured women’s voices the most (Sanders and Cuneo, 2010). The team process has created an “interpretive zone,” where multiple opinions were shared and discussed in order to encompass the important topics noted in each interview (Wasser and Bresler, 1996), and across all interviews (Sharp et al., 2019).

An index book was formed after consensus was reached on the major themes and sub-themes, and assigned different colors for differentiation purposes. Afterward, each team member analyzed each interview independently line by line. In group discussions, codes were compared line by line and differences in the analyses were discussed until concurrence was reached. When disagreements occurred (Gerstl-Pepin and Gunzenhauser, 2002), the majority who agreed on a specific code convinced the rest of the team, which followed with the accepted analysis, regardless of their seniority or academic status (Wasser and Bresler, 1996; Erickson and Stull, 2011).

**FIGURE 1:**
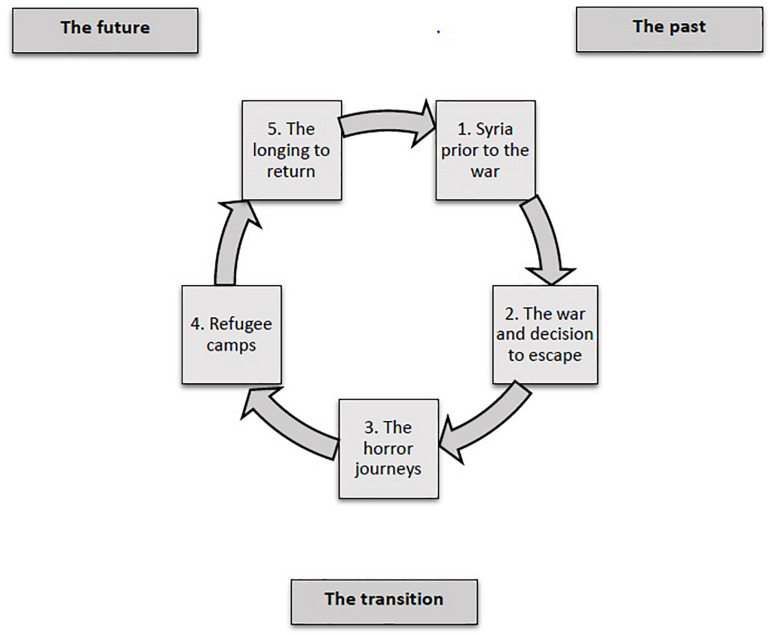
Themes of Syrian refugee women’s experiences.

Themes were divided into four major chronological phases: past, transition, present, and future, as well as sub-themes. The index book was changed and elaborated throughout the analysis process with the intention to remain true to women’s narratives (Clandinin and Connelly, 2000). The “past” included women recollections of their lives before the war, as well as during the war, followed by the decision to escape. The “transition or horror/death journeys” included internal displacement, the escape journey, refugee camps, and events preceded their arrival to the urban areas of Jordan. The “present” showed diverse difficulties faced as displaced refugees in Jordan. The “future” included their aspirations and hopes, which were manifested in their longing to return back home to Syria. This article will focus on women’s lives before the war, during the war, and the decision to escape; the transitional phase and hopes for the future (Figure 1). Details on traumatic experiences during the war and post exodus period challenges in the host country were discussed in another manuscript (Rizkalla et al., 2020a).

The process of forming the research team, translation, transcription and editing of interviews, data analysis and reaching consensus on the final analyses was completed within four and a half years. This long and slow process encompassed many challenges, among which the secondary traumatization (Figley, 1995) of the team members, who needed some distance and periods of separation from the materials in order to be able to return working on them.

## Results

### Sample and Participants

This study consisted of 24 Syrian refugee women who lived in various urban areas of Jordan; Amman, Irbid, Ar-Ramtha, Al-Zarqa, and Hiteen. Prior to fleeing Syria, women resided in Aleppo, Homs, Dara’a, Idlib, Sibenyah, Al Moadamyeh, Yarmouk Camp, and Damascus/Aleppo/Dara’a countrysides. Residency in Jordan ranged from 8 months to 5 years (*M* = 18.95 months, SD = 13.13), wherein they lived with one to nine family members (*M* = 5.04, SD = 2.34) in the same household. Ages of the women ranged from 21–55 years (*M* = 37.22, SD = 8.91). Marital status was as follows: 87.5% married, 8.3% widowed, and 4.2% divorced. Number of children ranged from zero to eight children (*M* = 4.29, SD = 2.07). In terms of socio-economic status: 70.8% were unemployed, and 56.5% reported their spouses were unemployed; 66.7% described their economic status as very low, and 29.2% as low. The vast majority of the women were literate (72.2%), and 64.3% had limited schooling.

Five women (21.73%) were injured during the war, and 30.4% had family members who were injured. Accompaniment during the escape from Syria to Jordan varied: 29.2% of the women escaped only with their children, 33.33% escaped with their husbands and children, 25% escaped with their children, husbands and other family members, and one woman escaped only with her husband, whereas two women escaped by themselves. In terms of entry to Jordan, more than half (75%) of the women crossed the border to Jordan legally, while 25% entered Jordan illegally, frequently via smuggling. More than half of the women (62.5%) resided in refugee camps before transitioning to the urban areas of Jordan, whereas 37.5% of the women did not reside in refugee camps. Of those who stayed in the camps, the average time spent there was 40 days. The shortest stay was for 2 days, whereas the longest period was of one woman who stayed for a year. A fair amount of women (54.2%) had family residing in Jordan prior to escaping. However, 45.8% of women, did not have family in Jordan, making such a transition more challenging.

### The Past

#### Syria – Prior to the War

All women recalled their lives prior to the war with a sense of nostalgia, regardless of their reasons for fleeing. They remembered Syria fondly, when they enjoyed freedom to travel, a calm life, social support systems, and in some cases, economic prosperity. Sahar described her freedom in Syria, which was interwoven with feelings of security, stability, and social connections: “There was security, there was stability… and we were loved by the people around us… I settled down and I did everything that I wanted with my own hands. I created inside my house everything that I dreamed of… I was really happy” (age 48, from Dara’a, 1.5 years in Jordan). The sense of security and pride in owning a house and a home was repeated in Sirene narration “It was, I swear, great [Mashala]. Thank God everything was provided, we had everything… He [husband] used to work and secured for us a home in Syria” (age 35, from Aleppo, 2 years in Jordan).

Though other women did not enjoy this same comfort, they were still nostalgic about their previous lives. Their recollections were suspended between the past and present. As Raya narrated “We were sheltered, I can’t tell you we were over the clouds and such, but we were financially comfortable” (age 36, from Aleppo, 1 year in Jordan). Nora also described less than an optimal economic status, however, these minimal conditions were sufficient in making her joyful in owning some independence: “We didn’t have anything except some land, trees, and a lot of plants [vegetables]… [Cheerful tone] I would grow flowers all around the house [chuckles]…” (age 41, from Dara’a, 2 years in Jordan). Yet, other women could not linger on nostalgic recollections from the past, since the suffering of displacement in the present was too painful to ignore. The following accounts attest to the inability of women to live peacefully in the present while their memories were shattered and connections with the past were sundered. Yasmeen said “We had an olive grove, a land, I mean, what can I tell you? We were, and we are here now and we don’t worth a thing” (age 39, from Dara’a Countryside, 1 year in Jordan). Amell also could not ignore her previous life when describing “It’s true we weren’t feeling much comfort, but we saw a lot of things that compared to now, we were relaxed. We were living in our own house and nothing bothered us, nothing” (age 22, from Al Moadamyeh, 1 year in Jordan). Losing their homeland and homes made women feel worthless, restless, discomfort, and faced difficulty in adjusting to the host country, which kept Syria even more memorable.

#### Living With War

Women voiced experiencing many atrocities during the war that affected them, their families, their larger community and physical surroundings. Once the war erupted, everything changed. Rania described the uncertainty and horror caused by the war “There was bombing and shelling, and we just had to leave. We didn’t know where we were supposed to go” (age 29, from Aleppo, 2 years in Jordan). Siham crafted her story around how safety was ruptured and fragmented in her close environment, which was replaced with fear for her own family “They would invade the neighborhood and kidnap kids. They kidnapped our neighbor. He was on his way to work… Then my cousin passed away and another cousin was imprisoned and everything fell apart outside. I feared for my kids, and then my husband told me ‘that’s it we should leave”’ (age 38, from Homs, 1 year in Jordan). Extreme violent acts were inflicted upon women and their larger communities in the aim of spreading fear and silencing the uprising. As mothers, women were mainly concerned for the safety and security of their children. Maha narrated on the toll taken from people around her due to their political involvement, which eventually resulted in losing her closest social network “All my friends left and those who did not leave were martyred by being tortured. I have friends who have experienced a lot in prison. Humiliation, and they all left. No one stayed in Syria” (age 29, from Al Moadamyeh, 9 months in Jordan). Other women were deprived of basic rights and faced many losses, among which were the ability to provide assistance, and autonomy to practice their professions: “Since I’m a nurse, relatives of my husband would bring to us the injured and I medicated them and they died right before our eyes” (Lina, age 40, from Damascus, 1 year in Jordan). Lina could no longer help the injured and rush them into the hospital, due to the Syrian regime’s surveillance, violence and control. She thus attempted less “provocative” acts of domestic medicine, which were doomed to fail. The regime perceived assisting the injured and wounded as acts of resistance that needed to be silenced by haunting, imprisoning or torturing the helpers. Such shifts in definitions from assistance to threats imposed on the national security have incapacitated women from providing womanhood nurturing methods and attending to their communities’ needs, as part of their active participation in times of conflict.

#### The Decision to Escape

Initially, not all women wanted to leave, but the exacerbation of violence has ultimately forced them to flee. For some, the decision to escape was gradual, while for others it was immediate due to the urgency of protecting themselves and their families. Hanan described “We didn’t want to leave the country. My mom, my kids, we weren’t going to leave… But we were scared for our kids” (age 48, from Damascus countryside, 1.4 years in Jordan). Women witnessed others fleeing and knew they should follow: “All the people and my neighbors, they all emptied their houses and left” (Muna, age 50, from Sibenyah, 1 year in Jordan). Nabila conveyed that the urgency to escape was influenced by rumors of others leaving “You kept hearing about the people who went to Jordan… went to Lebanon. I said you are already more familiar with Jordan, so better a place you know than getting to know another place” (age 42, from Yarmouk Camp, 8 months in Jordan). However, for some women, after gradually being convinced to flee, fleeing was complicated. As Amell articulated “People started telling us to go to Dara’a… so we brought ourselves to Dara’a. We couldn’t believe it when the road opened up. When we came, the road was closed to Jordan” (age 22, from Al Moadamyeh, 1 year in Jordan).

Some women found it difficult to decide between leaving with or without their husbands. Others either escaped alone or accompanied their family members, often their own children. In contrast, some women were apprehensive about leaving at all. Ghada faced difficulty in convincing her husband to escape: “I would say we should go to Jordan and he said that he would rather die in his country than go to Jordan. He said he didn’t like it, that he couldn’t go. I kept trying to convince him for a whole summer… we daily had an issue about leaving. He would say that he wouldn’t let me leave [alone]… You live with fear and horror, but in the end I said enough and I wanted my parents to get me out” (age 29, from Aleppo countryside, 1.5 years in Jordan). Eventually Ghada was forced to escape with only her children. On the other hand, Muna refused fleeing for a long time, which caused issues with her husband: “I wasn’t going to go and leave my house. We argued a little and I told him [husband] to go… So, my husband left and we [her son and her] stayed behind… The important thing is that I’m with my son and my house. I was a guard for my own house… I didn’t want to lose my house” (age 50, from Sibenyah, 1 year in Jordan). Other women faced difficulty with the decision to escape due to the inevitability of leaving loved ones behind. Wafa narrated with agony and fear “My mind is with my sick mom [still in Syria], I am scared” (age 21, from Al Moadamyeh, 1 year in Jordan). Escaping for all women was a forced decision that encompassed losing their homeland, homes, communities, extended families, autonomy, and independence in the sake of protecting themselves and their nuclear families.

### The Transition: Horror or Death Journeys

#### Internal Displacement, a Scattered Nation

Many women thought that they were temporarily leaving their houses with minimal belongings, only searching for provisional shelter in neighboring cities. Nabila recalled: “We said we would stay at my parents’ house for about a week or a month and then we would return… If we thought this was going to happen we would have emptied our homes” (age 42, from Yarmouk Camp, 8 months in Jordan). This temporarily escape has soon shifted to internal displacement as Hanan described: “There [Syria], we traveled from place to place depending on where it’s safe. So we’d escaped three times and stayed for 20 days. We’d escape at night. If you see our place, it’s like Day of Judgment. Everyone leaves. In cars, motors, scooters. Everyone with their family would leave. Escape to suburbs” (age 48, from Damascus countryside, 1.4 years in Jordan). However, internal displacement did not provide the safety women were hoping for. As Rula narrated “We left from one war zone and came to another war zone. Over there in that city [Aleppo] there was fighting and every day the airplanes were over us dropping bombs in Idlib too. There was shelling in Idlib… So we went from city to city and we started entering cities that we don’t even know” (age 32, from Aleppo, 1 year in Jordan).

Some women faced additional challenges and concerns, especially because they were women. For instance, violence has disrupted women’s ability to give birth safely, as Amell, who was approaching full term pregnancy articulated “I was very worried because there was no hospital… If you go somewhere in a car or take a bus and go to the hospital, there are a lot of checkpoints. If I would have reached the place after all of that, the child would have been dead inside me or I would have delivered on the way” (age 22, from Al Moadamyeh, 1 year in Jordan). Many escaped to different locations, however, the final result was a scattered nation, displaced inside and outside of Syria, which was perceived as especially painful to mothers and daughters. As Wafa narrated “We stay scared about my mother… She does not know anything about her kids. One is in Al Qunaitra, one is in Lebanon, one is in Dama, and one is in Moadamyeh” (age 21, from Al Moadamyeh, 1 year in Jordan). Hanan also described her own scattered family: “I have here [Jordan] three boys and a daughter. And one in Syria, and one escaped to Lebanon with her husband” (age 48, from Damascus countryside, 1.4 years in Jordan). For women, this new scattered situation was experienced as traumatizing and anxiety provoking in the personal and collective levels due to violations of their rights for safety and security.

#### Legality and Illegality of Escape

Some women escaped Syria legally, while others needed to be smuggled into Jordan to flee the destruction of their lives in Syria. For all women, escaping was accompanied with crossing checkpoints, being questioned and threatened, and facing the uncertainty of their journey. Hanan described the attitude needed when confronted with the Syrian regime during her escape, even when exiting Syria was conducted legally, “I came legally. You have to be calm in security; you have to be calm in front of them when they ask why you’re going to Jordan or what you’re going to do in Jordan” (age 48, from Damascus countryside, 1.4 years in Jordan). Muna narrated how questioning at the borders needs to be convincing despite its legality “Why did we get out in a lawful way? Because I told them that my husband was here [Jordan] and that I wanted to put my kids with their father, and I wanted to be with my husband. So they let me in” (age 50, from Sibenyah, 1 year in Jordan). Despite the legality of escaping and appropriate attitude, exiting Syria was not guaranteed and depended at times on the mercy of the officers at the borders. Elyanna conveyed a different narration due to the illegality of her escape “We went from Halab [Aleppo], spent a night in Damascus, and went to Dara’a. Of course we went illegally–without passports–just our clothes. They took us from one place to another. Suffering and struggling. They moved us three places until we got to Za’atari” (age 35, from Aleppo, 11 months in Jordan). Again, women courageously faced gendered challenges in their attempts to protect their pregnancies and infants. Yara described the risk of another woman who was smuggled together with her “She was pregnant, she escaped from Syria via smuggling and because her belly was big, they put many bags on her so they would never know that she was in the car” (age 28, from Homs, 1 year in Jordan).

#### The Death Journey – A Forced and Violent Escape From Syria

The escape journey included harsh weather, high expenses, extreme physical threats, checkpoints, scrutinization, and physical suffering. Siham portrayed the extent of danger during the death journey as follows: “It’s been a path of death. It’s like you were walking in the open dessert, this is how it was. It’s either you live or die, depends on your luck” (age 38, from Homs, 1 year in Jordan). Nora talked about the threat and physical suffering saying: “They walked us over to our destination, it took about an hour and a half. We walked among the trees and thorns” (age 41, from Dara’a, 2 years in Jordan). Moreover, the weather imposed further difficulties to the journey, especially for mothers: “The morning in that area was cold, it was winter of December-January and I’m in my heaviest coat. I have my two kids with me, the two kids were exhausted from the cold… I experienced cold in Syria, but not like that. I had to take care of my sick son, and the other one too” (Ghada, age 29, from Aleppo countryside, 1.5 years in Jordan). Dyala described the journey’s cost and other obstacles “We stayed for one week on the road until we got to Za’atari… Yes, each one 20,000 Syrian money… it cost us to get here [smuggling]… No, we only had a small bag. Only passports, and the family book, and the papers proving property ownership in Halab [Aleppo]. I lost them… I had passports but I lost them in the car” (age 55, from Aleppo, 5 years in Jordan). The regime inflicted violence against its escaping citizens and all women experienced fear and horror similar to Rania who depicted, “We were holding our hearts. They made us get off [bus], but I stayed… They threatened us by breaking the windows of the bus and a bullet came in from here—and came out here—right next to me. The bus was hit by bullets, and oh, the women and men were throwing themselves like this [on the ground] inside the bus! They didn’t leave any glass unbroken” (age 29, from Aleppo, 2 years in Jordan). Rula encountered similar threats and interrogations by the regime army “Every 25 meters there was a checkpoint and they would ask me questions” (age 32, Aleppo, 1 year in Jordan). The journey continued being extremely dangerous and life-threatening even after women left the populated areas of Syria. These threats were not only caused by the regime, but violence and terror were also inflicted on escaping Syrians by the Free Army and other fighting groups in abandoned areas. As Sahar narrated “They shot at us and surrounded us with their cars. We were driving through the desert without any lights—it ended up being the Free Army… They told us if the sun came up, they would shoot at us. The car was hit and they stopped to fix it while we were sitting on the hill waiting. It turns out that the Jordanians saw us and they signaled to us with a light that we should hurry up” (age 48, from Dara’a, 1.5 years in Jordan). Exposure to life threatening violent acts continued for women in the death journeys and traumatizing suspense while waiting to enter the borders of Jordan. The physical suffering during the journey was especially burdening for mothers as narrated by Farah’s experience, “We walked all the way from Syria to the borders… I had a small baby with me… She wasn’t allowed to cry, so no one would know that we escaped Syria illegally” (age 36, from Dara’a, 2 years in Jordan). Keeping a newborn’s silence during the journey is extremely traumatizing for a mother, as the gatekeeper responsible for the safety of the entire group of escaping Syrians.

#### Reaching the Borders of Jordan

Many women attested on the difficulty in entering Jordan when they reached its borders from the Syrian side. Navigating the correct entry to Jordan was also challenging, as Amell described “We were very scared of the checkpoint/border crossing, we couldn’t enter from the north… It was a lot of burden on us… hard. We don’t even know how we were able to reach Jordan” (age 22, from Al Moadamyeh, 1 year in Jordan). However, once given the title of a refugee, women consequently gave up on their right to re-enter their homeland, and return became dependent on temporary circumstances. Sirene explained how entering or exiting Jordan was uncertain: “I know some Syrians who stayed three months on the border… until they could enter Jordan… I can [go back to Syria and re-enter Jordan], but it depends on luck and border times” (age 35, from Aleppo, 2 years in Jordan). Escaping one’s own homeland and crossing borders encompassed anxiety provoking moments. Rula narrated “When I went to the first checkpoint, a big officer came up to me and I got really scared. I was pregnant and starting my 9th month. When I went in, I got scared, I started to shake from my fear. I didn’t know how to answer his questions. I’ve never in my life crossed a [country] border” (age 32, from Aleppo, 1 year in Jordan).

Women complained about the harsh treatment they received from the Jordanians upon arrival to the borders. Siham attested with anger and frustration, “At [Jordan’s] border… The Jordanian security, the attitude of humiliation is in perceiving us as stupid, I mean it wasn’t enough we got humiliated in our country, but it was so much easier than the way they treated us [worse than in Syria]… [They said] ‘Every one of you, take everything out from your bags and dump it onto the ground… all these things are forbidden [entry].’ They were yelling at us and humiliating us just like the ones we had [in Syria]” (age 38, from Homs, 1 year in Jordan). Uncertainty, humiliation and fear continued being profound components in women’s ordeal exposes during crossing the borders.

#### Refugee Camps

After overcoming the horror journeys, women expected to reach a safe haven. However, they were surprised that the main refugee camp in northern Jordan, Za’atari, inadequately accommodated refugees. The challenges started with entering the camp, registration and initial accommodation stages. Sirene conveyed the helplessness of refugees who reached the camp without adequate documentations “In Za’atari camp, a lot of them don’t have proof. A lot of them left without ID cards, family books [family lineage books] were gone… If you don’t have proof, you’re done” (age 35, from Aleppo, 2 years in Jordan). In the initial stage after entering the camp, refugees were placed in a big ‘welcome tent,’ which was perceived as far from being welcoming or adequate for women and their children. As Elyanna explained “Group setting. Before they put us inside the camp, everyone sleeps together. Not just us—families and families… Toilets without doors. And we all sleep together—you don’t know where the people come from… There’s no police to keep you out. A man came inside… He told us ‘in Za’atari, expect everything. If you have a daughter, worry about your daughter. If you have money, worry about your money. If you have gold, worry about it’… We slept on the floor. They gave us blankets, those army blankets. I have rhinitis… We struggled a lot” (age 35, from Aleppo, 11 months in Jordan). Rula articulated how difficult this initial stage was: “I came immediately from Idlib to the Za’atari Camp… I was scared… I was pregnant and I had my 4 kids with me… I arrived at 4 am in the morning… They gave me yellow cards, nutritious food, and 4 cushions, I put them up like walls, like a tent, so that my kids and I can sleep in it until they can secure me a tent of my own… I’ve stayed for a week, and I couldn’t handle more than a week” (age 32, from Aleppo, 1 year in Jordan). Other women expressed how overwhelmed they were from the refugee camps and the atmosphere in them: “When we entered into the Za’atari, I felt as if I entered Abu Greeb prison. What is this? [You would see] sticks, wires, and the kids standing with carts waiting for pickups, and the cars. And 300 lira to rent a car? And a drive, they’d take you if they didn’t steal you first [they would]. And being catcalled [verbal sexual harassment] with knives? Oh waw, how great [bitter laugh]. I told him [husband], ‘Where did we come to? This is the Za’atari?’ Oh God! How was this the Za’atari? How graceful is the Za’atari [sarcastic]” (Siham, age 38, from Homs, 1 year in Jordan).

Women also described the chaos in the camp, lack of basic needs, difficulty accessing facilities, the harsh weather and hardships refugees experienced, which were further exacerbated by refugee destitution. The weather imposed a challenge during the summer or winter: “The day we came, it was extremely frosty, cold, and they had laid down mats, tiny mats inside of the tents. There was nothing” (Yasmeen, age 39, from Dara’a Countryside, 1 year in Jordan). Nora, on the other hand, described the impact of the desert climate in the summer as “our eyes also got really irritated [dust]. We would have to open one eye and close one eye… And it would get so, so hot in the tents. Oh, how hot it got! I would go crazy! You’d enter the tent and feel heat like fire” (age 41, from Dara’a, 2 years in Jordan). Many of the refugees’ needs were unaddressed. Sahar described how UNHCR finally provided her with a tent “Yea, but there’s no sleep! Their mattresses were soaked” (age 48, from Dara’a, 1.5 years in Jordan). Access to facilities, services and food were also challenging, as Amell articulated “If you want to go to the bathroom you have to wait in the lines of people” (age 22, from Al Moadamyeh, 1 year in Jordan). Hala described the long wait and panic of refugees at the camp food distribution point saying, “I would go from 6 o’clock in the morning just so I can get 1 kilo of rice or 1 kilo of groats or 1 kilo of lentils for my kids… And when they open the door for distribution, the people waiting would fall on top of each other” (age 30, from Homs, 1 year in Jordan). Women feared the general atmosphere in the camp and found it difficult to witness other refugees’ miseries: “The tragedies, the things people at the Za’atari saw, you could feel that” (Ghada, age 29, from Aleppo countryside, 1.5 years in Jordan). Nora expressed how the camp’s conditions were less than minimal and how the suffering of others impacted her: “We stayed up until morning, sitting and sleeping in the cold. I’d see all the women and children, all the sadness and oppression around me. How could I sleep after seeing that?… They gave us blankets. But it wasn’t enough. My little girl… She didn’t even want to use the bathroom there, because they weren’t clean or sanitary…” (age 41, from Dara’a, 2 years in Jordan).

In addition, women did not feel safe in the camp, and had to be very cautious in taking care of their children. As Nora attested “I count my children. I have to, because there’s a lot going on. My brother’s wife lost her daughter” (age 41, from Dara’a, 2 years in Jordan). Other violent acts and threats have also put women at risk, as Siham narrated “They robbed me of my belongings… They seem like the type that is wicked… they looked like undignified, with knives, and have scars, like sights that doesn’t let you feel at peace, and their attitude is not good as well” (age 38, from Homs, 1 year in Jordan). Sexual harassment and other privacy violations imposed additional threats on women and girls’ safety. Hala described “There is a possibility that men go inside the women’s bathrooms. They will enter as disguised to women with muffler dress and go inside the women’s bathrooms” (age 30, from Homs, 1 year in Jordan).

The difficulties in the camp were intolerable for women, to the extent that they could not bear staying more than a few days, or even paid bribery to get out. Yasmeen admitted with concern: “They have illnesses of infections, and the sickness of I don’t know what, I mean, life is really, really hard in the camp, I couldn’t stay with my kids there” (age 39, from Dara’a Countryside, 1 year in Jordan). Siham was anxious since her daughter was sexually harassed in the camp: “I told my husband ‘It’s impossible to leave Rawan [daughter] here for the night. We have to get her aunt to pick her up. Right now, right now, you’re going at this moment to approach any of the officers here and tell them to get my daughter out of here, now”’ (age 38, from Homs, 1 year in Jordan). The difficulty to access facilities, corruption and inadequate basic needs have also contributed to escaping from the camp. Yasmeen expressed “There was no clean water, and everything was far away, I mean we could not live in the camp… everything is possible with bribes… They smuggled them out from the Za’atari camp… they bribed them again… God I would rather sit in the street or go back to the shelling than to stay in the camp” (age 39, from Dara’a Countryside, 1 year in Jordan). Nora was also smuggled with her family out of the camp, saying “We paid, yes we paid [to get out]… we ran from the Za’atari” (age 41, from Dara’a, 2 years in Jordan). This dreadful experience in the camp was perceived as humiliating, to an extent that some women regretted escaping their homeland. As Siham articulated with agony “In Za’atari, it isn’t even a normal life to live. I spent 4 days in Za’atari… Why did we leave our Syria? It was better to die there with our dignity than being here” (age 38, from Homs, 1 year in Jordan). Due to the harsh conditions in the camps, malnutrition, humiliation, sexual harassment, and threats to their lives and their children’s lives, women were forced again to find the means to escape from the camps.

#### The Difficulty of Staying in Jordan – Back and Forth

Women recounted diverse situations in which they needed to return back to Syria after the initial entry to Jordan as refugees. Women had to make arrangements for their children or assist with their parents. Still, these rapid re-entrances were uncertain and imposed some risks. Muna needed to re-enter Syria for her children’s safety “I’m scared for myself and my kids. I had to go between all the fighting in order to get my daughter… Yea, I got them out [her daughter and son]. I put them there [in Jordan with their father], then I went down [to Syria] with the youngest” (age 50, from Sibenyah, 1 year in Jordan). Siham who was preoccupied with her sick elderly mother and needed to get back into Syria for her, attested “My mother told me, ‘Dear daughter, if you are going to leave you won’t be able to come back.’ I told her, ‘No, with God’s permission I’ll come back. I will take the risk on myself, I will come and go and travel, there’s no problem.’ I’m not afraid of anyone, thank God [Ilhamdola] except from God” (age 38, from Homs, 1 year in Jordan).

Other women conveyed that their spouses left them in Jordan and went back to Syria as the shelling was still happening and the situation looked bleak, because they could not tolerate living outside of their country. Nabila narrated that her husband was determined to return but she was not sure of his success “If God is willing, on Sunday or Monday, he is going back to Sham [Syria]… No. I told him, returning, I’m not going back. I am not going back with him [shivering voice]… It’s 50/50, either they let him go back or they don’t let him go back. Most of them, they don’t allow to return” (age 42, from Yarmouk Camp, 8 months in Jordan). Yasmeen’s husband had also left due to the hardships in Jordan “He came here to be with us. After some 4 or 5 months, he saw the conditions here, he worked here, he worked there… The salary is low, there is no… He took himself and went back to Syria. He has been there for 6 or 7 months” (age 39, from Dara’a countryside, 1 year in Jordan). Hala claimed that Syrians left Jordan due to the toll they experienced, which was justified in her opinion “There are a lot of people, I swear to God [Wallah], the people who I know the most have returned to Syria… I would rather be humiliated in our homeland, our country, than be humiliated in here” (age 30, from Homs, 1 year in Jordan). Again, the broken pride of Syrians who tried to survive in Jordan, and the humiliating conditions they felts as unwelcomed refugees had convinced women of their and others’ necessity to return.

### The Future and the Longing to Return Home

For many, the journey was not over. Each woman expressed yearning to return home to Syria. Some women thought their displacement was temporary and did not realize they would stay in Jordan for years. “We thought that we would be gone maximum one month, then return home” (Sahar, age 48, from Dara’a, 1.5 years in Jordan). Other women articulated that returning back home is dependent on the political conditions in Syria. Though women did not express their political and national ideologies openly, but rather focused on their personal and gendered stories; patriotism and attachment to their homeland were illuminated with their persistence to return and resistance to stay in Jordan. As Hanan articulated her sense of uprootedness and homelessness “We just want things to calm down at home so we can return. What do we have here? Our life is there. To return to my country—I have nothing but that wish” (age 48, from Damascus countryside, 1.4 years in Jordan). Sirene also expressed that returning is related to her roots, and family connections “First and foremost, I want to return home… If Syria gets better today, the next day I’m going. If today there’s nothing, I’m going tomorrow. One belongs next to one’s family. To see her sister. For her father to hold her… that is worth more money than anything in the world” (age 35, from Aleppo, 2 years in Jordan). However, women wanted the grantee of safety before undertaking such journey back, “If I said no [to wanting to return], I’d be lying to you. I swear. If all permits, with God’s will [Inshalla]” (Dyala, age 55, from Aleppo, 5 years in Jordan).

Elyanna also narrated her wish to return and for her country to be safe again “I want to go back to Syria… For my country to return the same. We used to live okay. Everything that was messed up, it wasn’t ours” (age 35, from Aleppo, 11 months in Jordan). Despite Elyanna’s wish to return, she was convinced that the consequences of war were not the people’s responsibility to blame for and that returning encompassed political measures. Other women acknowledged that returning would take a personal toll and losses, as Nisreen attested that if she returns, her home will not be there anymore “To return to Syria—not to my home… When things settle, of course I want to go back to Syria” (age 46, from Aleppo, 4.5 years in Jordan).

Some women viewed their return home as a realistic endeavor, while others saw no possibility for return. Nonetheless, all lived in uncertainty regarding to their situation. Muna had a wishful thinking that her home would not be demolished when she returns “I left by myself with the belongings I had from my family’s house. Until now, I have hope that God will protect our house and that we will eventually return to it” (age 50, from Sibenyah, 1 year in Jordan). Nora was more realistic in her hopes, saying “But the houses got robbed, we don’t have any more belongings in Syria… I dream that everything would go back to the way it was. That I’m ensured, that my kids can walk to school safely. And for them to come back to a home. That we have our own house” (age 41, from Dara’a, 2 years in Jordan). However, Amell who knew she cannot return because safety and security were threatened, narrated “Now if we returned, how would we return? We don’t know… Like we are like this, living and wanting to return. But if it doesn’t work, we aren’t going to return” (age 22, from Al Moadamyeh, 1 year in Jordan).

Still, being in one place while dreaming and longing to be in a different place can impose a challenge and make adjustment to the new country more difficult. Experiencing displacement and having family members scattered can take a toll. Yasmeen spoke about the adjustment challenge with agony “The thing is now, I am setting up in a house, and I am building a nest, while I just want to go back to my house, to my country… We will go back to our country” (age 39, from Dara’a Countryside, 1 year in Jordan). Farah who clung to her country and familial roots said, “I won’t agree that my daughters marry someone from Jordan, because I don’t know when we will return to Syria… I want my daughters to stay around me, I don’t want them to marry strangers” (age 36, from Dara’a, 2 years in Jordan). Women articulated resistance to adjusting to the host country and new status and persisted in their yearning to return to their homeland.

Some women attributed attachment to their country, patriotism, political, and ideological aspects to their longing to return, mixed with a sense of astonishment from being displaced and thus refusing to accept their new identity as refugees. Sahar narrated “I didn’t expect all this to happen to us… I’m nothing now” (age 48, from Dara’a, 1.5 years in Jordan). Hala suggested political solutions to enable Syrians to return “Instead of taking us overseas, restore the security, so we can return to our country” (age 30, from Homs, 1 year in Jordan). Siham spoke in patriotism about her attachment to her country “What should I tell you? First, we didn’t come here willingly. It’s almost impossible that we would leave [the country]. The country [Syria] is very precious to us” (age 38, from Homs, 1 year in Jordan). Yara expressed the pain of being a refugee: “When someone says, ‘With God willing [Inshalla] you will get back to your country,’ this phrase is hard [painful]… It’s hard because I can’t comprehend that I left my country!” (age 28, from Homs, 1 year in Jordan). Women’s collective suffering, pain, and agony of being uprooted, transforming them into refugees had reinforced their regret of escaping and intensified their longing to return.

Many women dreamt about going back to their homes, the same homes they left and imagined how they are going to actively fix and rebuild them: “Once Syria returns, the house will return to us… Yea, like we’d fix the walls—and make everything new again” (Nora, age 41, from Dara’a, 2 years in Jordan). Women also dreamt about gaining their previous lives back, and hoped for a better future for themselves and their families. “I hope that I return… To a life that is better than the first one. That everything that is happening right now, everything, everything goes away and ends. That I can do everything for my kids and secure their future” (Amell, age 22, from Al Moadamyeh, 1 year in Jordan). Farah recalled one of her dreams with disappointment and despair since she knew it is not going to be fulfilled, “I dream about being in Syria that I see my neighbors and relatives, but then I wake up and find myself here” (age 36, from Dara’a, 2 years in Jordan).

However, others kept Syria as only an agonizing dream that is far from being achieved. “We don’t know when we’re going to go back, when these problems will be over, when this oppression will be over, if we will go back to our country or if we won’t go back, Syria is now nothing but a dream for us” (Rima, age 50, from Al Moadamyeh, 1.5 years in Jordan). Rima, like other women acknowledged that only an end to the current political power, violence, control, surveillance, and oppression will enable a safe return to their homeland. Until such political solution is accomplished, the yearning to return will only remain a shattered and unfulfilled dream.

## Discussion

This study examined Syrian refugee women narrations that looked back on their lives before the war, during the war and the transition phase of the escape journey, and their future hopes and aspirations to return back home. The importance of this study lies in its representation of women’s uncounted narratives as they voice personal and gendered traumatic recollections, which illustrate a broader memory of refugee denied collective suffering (Shalhoub-Kevorkian, 2010). As Sayigh (1998a) claims: “Essential to the physical and cultural reproduction of collectivities, women members reflect and contest ideologies binding them to gender-specific tasks and roles. Women’s personal narratives, whether written or oral, mono or polyphonic, structured story or fragmentary testimony, have value in illuminating this contested subjective domain that national and social movements repress” (p. 170). Regardless of how the women lived in Syria or the challenges faced in Jordan, they all looked back fondly on their situation prior to the war and were yearning to return to Syria. Refugee women discussed life before the war (Gorst-Unsworth, 1998), accompanied with testimonies of deep nostalgia, happiness, and comfort of having a home, security, an identity, a country, a united family, and support systems. However, their safe and comfortable life, even for the majority who had scarce resources, had shifted when the war began, and became one that was rife with traumatic experiences.

Women endured many tragedies and traumas in their home country during the war (Rizkalla et al., 2020a). Syria became a war-torn place with killing and destruction for many women (Adwan, 2010), which led to the decision to flee. Many witnessed death, were subjected to physical and sexual violence, torture, and other atrocities (Gerard and Pickering, 2014; Farhat et al., 2018; Rizkalla and Segal, 2018, 2019b). What they remembered as the Syria they knew, no longer existed once the war erupted, and collective loss took over, as it was engraved in Sahar’s phrase: “Our country is ruined” (age 48, from Dara’a, 1.5 years in Jordan).

For many, to escape and leave everything behind was not an easy decision, especially because they knew that such escape would encompass a horrifying journey with irreversible consequences. Leaving all that they knew to an uncertain future was a decision that meant life or death for the majority of women, “every time we wanted to leave, we felt as if one foot is going forward and the other foot is going backwards [hesitant]” (Nabila, age 42, from Yarmouk Camp, 8 months in Jordan). Such forced decision of dispersion and land loss illuminate gendered experiences of mobilization intertwined with insecurity and anxieties (Sayigh, 1998a).

The title of the book alone “We Crossed a Bridge and It Trembled” (Pearlman, 2017) provides a glimpse on the risk that Syrian refugees undertook during their escape journey. The journey was often violent, “There started the shooting from the left and right. I don’t know what the area was called, but we got eventually… into the bus… The drivers would tell us: ‘Don’t tell them [the guards at the checkpoint] that we’re going to Jordan’… We told them that we’re going to visit our relatives in Sit Zainab” (Siham, age 38, from Homs, 1 year in Jordan’). The silence, denied recognition, and invisibility of suffering during their journeys reveal women’s “ordinarily unheard voices that contest the normalization of violence in conflict zones… [which] raise new sets of questions that revolve around acquiring justice and alleviating the pain of those living the ‘everydayness’ of militarization and violence” (Shalhoub-Kevorkian, 2010, p. 7).

Escaping for many women was conceptualized as a temporary solution for the sake of protecting themselves, their children and families and expected to return fairly quickly, “I didn’t even put locks on the door [of the house], I just closed the door because I knew that I was going to come back” (Muna, age 50, from Sibenyah, 1 year in Jordan). However, regardless of the urgency to escape, or calculated process and preparations made before leaving, all women faced daily upheavals and were forced to escape under fire, shelling and constant fear of not being able to remain in peace and safety in their homes and neighborhoods.

Journeys for some women started with internal displacement for a portion of time and diverse housing arrangements after their homes were demolished (Verme et al., 2015). In October of 2014, every governorate in Syria had internally displaced people, to an extent that some governorates had almost half of their population being from a nearby town or village (Doocy et al., 2015). For many women internal displacement took the form of one or many cities before leaving to Jordan. Women reported that a Syrian could be internally displaced as much as a total of three times before leaving Syria to a host country.

Some have described escaping legally from Syria, whereas others were forced to be smuggled illegally, through means of forging and bribery, after attempts of legal routes were denied. Regardless of the methods used, all women articulated taking a great risk while escaping under shelling and other threats, such as being caught by the Syrian regime, the Syrian Free Army or other armed groups. Many women were able to find ways to escape Syria illegally to ensure their families safety, and the vast majority spent some time in refugee camps. The struggles embedded in their journeys testify to the strength, courage, and resourcefulness of women as representations of personal and collective suffering and resilience (Sayigh, 1998b).

However, with the hopes of reaching a safe haven in Jordan when finally crossing the borders, women were astonished to discover that the horror journey has not ended, which made the refugee camps a transitional phase for a more reasonable move to Jordan. The experiences in the refugee camps were beyond their capacities to tolerate and imposed new threats to their lives and safety. In the camps, women experienced being taunted by Jordanian officials or other Syrians, corruption, drugs, prostitution, fear for their daughters from sexual violence, and the hard living conditions in tents that could not withstand the harsh weather conditions (Jabbar and Zaza, 2014; Barkil-Oteo et al., 2018). All women escaped the camps and could not stay there long. On average the women stayed in the camps for 40 days, with only one woman reported being forced to stay for a year. Again, women described illegal methods to escape the camp via smuggling or infiltration, if a legal route was denied (a relative in Jordan sponsoring their exit).

After reaching the urban areas of Jordan, many women described starting a new journey, although less horrifying, but not less painful as displaced refugees in exile. They all faced diverse difficulties such as poverty, housing issues, unemployment, unmet survival needs, lack of support systems, and locals’ hostility, as well as physical, mental, and somatization sequelae (Rizkalla et al., 2020a). These acts of violence—the attacks on the body, the home and homeland, which work in a spiral manner—termed as “spiral transgressions” made many Syrians homeless, but they have also disrupted women’s rights to safety, and violated their access to education, healthcare, and social networks, reflecting their wide-ranging consequences (Shalhoub-Kevorkian, 2010, p. 6). Though Syrian refugee women have sought refuge in Jordan, it served as their host country but nothing more; they did not call Jordan home. Thus, “home” is not only seen as a physical site, but also an emotional space, emphasizing women’s survival, resistance and agency, where they can feel safe, humane and dignified (Shalhoub-Kevorkian, 2010).

Women articulated in great agony that their new titles and status as refugees and the uncertainty that it encompasses were very painful and stressful to face. Yara explained that it is more than just the land that defines what being a Syrian is; “I wish we could just return to our country, at least we could say, ‘I am Syrian’ and you could be proud of yourself. At least, you stay in your country and no one interferes with your business, these are the simplest things” (age 28, from Homs, 1 year in Jordan). The historical and political rupture of the Syrian crisis revealed in women’s narratives had an effect on the rupture of women’s personal identities and their representations of the self, which reflects as well a collective identity crisis (Sayigh, 1998a).

Women also acknowledged how scattered and fragmented the Syrian nation became, their society, communities they once knew and lived in, and their family members who were struggling in different countries, or the ones who were still in Syria and they constantly feared for. They were all uprooted, stateless and scattered in different geographical locations, with unseen prediction upon reunion. Separation from family members was found as a traumatizing event (Rizkalla and Segal, 2018). The issue of separation and desire to be reunited was shared by many women, “my memory is still attached to Syria, to Homs. I have my sisters over there. I am scared for them a lot, there isn’t any food, and no water. [Short pause]. Like I have a brother, one has nothing other than one’s siblings, one’s people, and one’s family [shivering voice]” (Hala, age 30, from Homs, 1 year in Jordan). Narrations were gendered due to women positioning themselves as mothers, sisters, and daughters, rather than as national subjects. The national crisis was measured by their painful separation from family ties and disconnections from parents and children (Sayigh, 1998b).

Though Jordan has generously hosted the Syrian refugees and humanitarian organizations had the best intentions to provide their needs, many refugees are still facing challenges (Rizkalla and Segal, 2019a, 2020), especially economically (Miller and Rasmussen, 2017; Walton, 2018). For many women who enormously struggle on a daily basis in Jordan, the one thing that they aspired for and gave them hope to survive, regardless of how tangible the aspiration was—they all longed to return home—to Syria. Many women yearned to return and restore whatever is left of the Syria they once knew and aspired for a better future than before.

The shattered dream of a better Syria that has initiated the war, seemed for some as far away from being accomplished, however, “return” was tangled with political ideology and activism needed towards achieving it, “we are going to return, meaning that at the end, we will come back to our country and rebuild it and do things… But unfortunately the situation that is happening in the country is very concerning… I feel this nostalgia and this longing that I have to return to Syria… but maybe I could do something for the future [from within Jordan], that if I go back to the country then things will be better” (Maha, age 29, from Al Moadamyeh, 9 months in Jordan). Although the Syrian people yearn to return, instability and threatened safety in their country have deprived them from the possibility of return. Thus, they are trapped. Returning back after becoming refugees would render them with a new title of “infiltrators” since they have dared to resist the regime’s surveillance, sovereignty, and silencing (Shalhoub-Kevorkian, 2016), and “voluntarily” deported themselves from their homeland. Yet, staying in Jordan took a great social and class degradation toll, as Hala described eloquently “Because seriously we have seen a lot here, we did not expect it would be to this degree. Exploitation of everything, in all aspects of that term” (age 30, from Homs, 1 year in Jordan). The implications of “rebellious” returnees as infiltrators and the incapacity to return have invaded all women’s intimate territories of life; their bodies, health, familial and social disconnections, and abandoned them to cope with an open wound, pain, oppression, abuse, subjugation, and uprootedness all by themselves (Shalhoub-Kevorkian, 2016).

Most women have voiced their war and escape challenges and were presented at times as disadvantaged objects of the harsh circumstances and rupture of individual agency who only wanted to protect their families and children. Nonetheless, they have also shifted in their narration into becoming subjects who made decisions, protected others and managed their households, entwined with taking a powerful active role in their longing to return to their homeland and participate in rebuilding it. These shifts in narration could be viewed according to Al-Ali(2018, p. 158) as “women’s reproductive responsibilities,”, in owning a political ideology and taking a social activism stand in regaining universal justice whilst wishing to contribute and reconstruct their country in the future. However, women’s participation in exile is marginalized and deferred until their homeland is liberated and peaceful and safe return is enabled. Though escape has mobilized women out of their country and into gender role changes, they still guarded their identities, family, and community traditions (Sayigh, 1998a). “Women tell history as witnesses of political events, as political actors whose participation changes over time, and as mothers/housewives/community members whose roles also reflect political, ideological, economic, and social change” (Sayigh, 1998b, p. 43).

Women shared that it was not solely them who felt this way, but also those who joined them on their journeys—their children and husbands. To an extent that some of them had relatives and husbands go back to Syria while the war was still taking place, leaving their wives and children behind, because they could no longer tolerate staying in Jordan (Barkil-Oteo et al., 2018). The children spoke of the need to return by sharing their constant hopes of going back home and asking innocently why they have not returned and why they cannot stay with family members in Syria (Jabbar and Zaza, 2014). It was clear that children articulated their wishes openly, mirroring the painful loss of their homes in front of their mothers (Rizkalla et al., 2020b).

Even after this long, treacherous and often life endangering journey that was outlined by women’s stories, the wish to return was explicitly present. Whether in an optimistic way when saying “Syria will return, and we will return to our country…” (Rania, age 29, from Aleppo, 2 years in Jordan), or in a realistic way of acknowledging that it is an unlikely scenario, “We are not going to return, and if we do return, we are going to eat a lot of gravel, like that’s it, it’s gone, there isn’t any Syria” (Maha, age 29, from Al Moadamyeh, 9 months in Jordan), there was a sliver of hope that return could be a reality. Steinbeck (1962) said: “You can’t go home again because home has ceased to exist except in the mothballs of memory” (p. 163). The home of the refugees is likely a physical rubble, its social networks no longer exist, and even if they did return, it will take years and many generations to rebuild what was violently destroyed.

The journeys that women undertook were so traumatizing and displacement challenges were intolerable to an extent that “building a nest” and “a home” in the new country is impossible. While their physical bodies were in Jordan, their mental existence remained in Syria, making adjustment in the host country harder to accomplish. Feelings of social isolation and estrangement have also contributed to women’s lack of adjustment, especially when they lack work opportunities and educational solutions for their children, mainly resulting in leaving them at home to care for the chores and child rearing (Rizkalla et al., 2020b). The cost of living in Jordan today is even higher than what it was in 2014, and imposes further adjustment struggles for refugees (Rizkalla and Segal, 2018). This lack of adjustment encompasses mental health consequences in maintaining anxiety from the unknown future and depression of being unable to make further changes to safeguard their wellbeing (Rizkalla et al., 2020a). When women’s hands are chained, with no capacity to stop the war, or start rebuilding their country, in addition to the dissatisfaction and continuous hardships faced in the host country, which even escalate with time, may deteriorate their mental health in providing a doorway to helplessness, hopelessness, and feelings of being trapped and broken. The only strategy to cope with the destabilization of Syrians lives, the politics of denied justice reflected in the invisibility of their suffering, and mental health consequences for women are their hopes and dreams to return back home someday.

### Limitations

There are few limitations to this study that need to be considered. The interaction between the researcher and women was not included in the analysis, which might have some impact on the narration. The interview setting at humanitarian organization could have influenced the interviewees in presenting a certain picture with the hope of gaining some benefits to address their urgent needs. In addition, the analysis did not include the interactive process of the research team, which may have benefited the analysis, especially since the majority of researchers were women, similar to the interviewees (Wasser and Bresler, 1996). Furthermore, the findings remain descriptive and do not permit absolute conclusions about refugee’s journeys and their longing to return. Each aspect of the journey could be studied on its own and the decision related to the beginning and end of the journey for each woman was not a simple endeavor (Benezer and Zetter, 2014), especially that each woman shared a unique understanding on phases of life, nostalgia, and storytelling. As a qualitative study that has a small sample size, it is further limited by the homogeneity of participants’ characteristics (i.e., women, refugees, etc.). Despite these limitations, the study presents an important perspective and a continuous overview (eagle eye) on the understudied topic of refugee escape journeys, and the toll they took from Syrian refugee women, and its relationship to their yearning to return back home.

## Conclusion

This study challenges the understanding of refugee flight by examining the process of the escape journeys as life-transforming experiences. Despite the high risks and life-threatening events in escaping from war-torn Syria, all refugee women longed to return home to their country. Studies on refugee journeys are scarce, making the knowledge on their lived experiences and public understanding limited, especially in regards to the concepts of “home” and “return.” The study may shed some light on the way we view refugees’ decisions to escape, routes, and hurdles when fleeing traumatizing conflict zones, and conditions in the host countries. This study also challenges the perspective on refugees, especially with the increasing xenophobia globally and the hostility in host countries, embedded in the fear of the new unfamiliar residents. Women who participated in this study intentionally wanted their stories and voices to be heard in the purpose of the world to gain a better understanding of their suppressed collective suffering and their yearning to return to their lost lands, homes, and country. Such revelation and verbalization of women’s unheard stories can reflect their resistance to injustices and preservation of humanity as political acts.

The Syrian war has been continuous for almost 10 years, with new political and offensive military operations still occurring everyday, making the dream of returning back to a peaceful home even more out of reach. By the end of 2014, 45.5 million refugees were displaced to countries not their own; 0.5% returned home or were resettled, whereas 6.4 million were in protracted refugee situations. As tragic and hopeless as it may seem, it would appear that most of today’s refugees are destined to remain in their host situations (Segal et al., 2018).

The longing to return home can provide an effective means of coping with the challenges of displacement. Nostalgia can help refugees deal with the separation and loss they have encountered after leaving their country and provide solace in coping with the hardships of uprootedness (Kallivayalil, 2013). Return was an inevitable wish, and “Home” both physically and emotionally, remained Syria for all refugees. Yet, it is a future that the world needs to help refugees secure. It needs to find plans for economic security, and stability that enable building an identity and a life renewed, even if the “new nest” is “temporary” while waiting to return home. Syria was a “paradise assaulted” by collective and institutional evil, and though the world witnessing the dehumanizing atrocities and suffering inflicted on its people en masse and may have the capacity to care, it still stands speechless. However, silence is complicity, as in “stealing the pain of others” and not transforming the outrage into taking responsibility and action directed against the abandoned Syrians’ injustices (Razack, 2007). Advocating for gender equality and societal change might eliminate gender disparities and reduce the challenges Syrian refugee women face in host countries. A policy change needs to take into account combining advocacy for human rights and the restoration of political peace and moral justice in the Middle East to enable refugees to return back home and for women to actively participate in reshaping their future.

## Data Availability Statement

The datasets presented in this article are not readily available because of their confidential and sensitive nature as they were collected from refugee women. Requests to access the datasets should be directed to NR at rizkalla555@berkeley.edu.

## Ethics Statement

This study was approved by the Committee for the Protection of Human Subjects, University of California, Berkeley (CPHS, February 2014). Written informed consent for participation was not required for this study in accordance with the national legislation and the institutional requirements.

## Author Contributions

NR and SPS: conceptualization and supervision. NR: data curation, investigation, methodology, and project administration. NR, SA, RA, and LS: formal analysis. SPS: funding acquisition. NM and NR: visualization. NR, SA, and NM: writing—original draft. SA and SPS: writing—review and editing. All authors have read and agreed to the published version of the manuscript.

## Disclaimer

The views expressed in the manuscript are the authors own opinions, and not an official position of the institution or funder that supported this study.

## Conflict of Interest

The authors declare that the research was conducted in the absence of any commercial or financial relationships that could be construed as a potential conflict of interest.
